# Clustering and Smoothing Pipeline for Management Zone Delineation Using Proximal and Remote Sensing

**DOI:** 10.3390/s22020645

**Published:** 2022-01-14

**Authors:** S. Hamed Javadi, Angela Guerrero, Abdul M. Mouazen

**Affiliations:** 1 Interuniversity Micro-Electronics Center (IMEC), Kapeldreef 75, 3001 Leuven, Belgium; hamed.javadi@imec.be; 2 Precision Soil and Crop Engineering Group (Precision SCoRing), Department of Environment, Faculty of Bioscience Engineering, Ghent University, Coupure Links 653, 9000 Gent, Belgium; angela.guerrero@ugent.be

**Keywords:** clustering, feature selection, management zone delineation, precision agriculture

## Abstract

In precision agriculture (PA) practices, the accurate delineation of management zones (MZs), with each zone having similar characteristics, is essential for map-based variable rate application of farming inputs. However, there is no consensus on an optimal clustering algorithm and the input data format. In this paper, we evaluated the performances of five clustering algorithms including *k*-means, fuzzy C-means (FCM), hierarchical, mean shift, and density-based spatial clustering of applications with noise (DBSCAN) in different scenarios and assessed the impacts of input data format and feature selection on MZ delineation quality. We used key soil fertility attributes (moisture content (MC), organic carbon (OC), calcium (Ca), cation exchange capacity (CEC), exchangeable potassium (K), magnesium (Mg), sodium (Na), exchangeable phosphorous (P), and pH) collected with an online visible and near-infrared (vis-NIR) spectrometer along with Sentinel2 and yield data of five commercial fields in Belgium. We demonstrated that *k*-means is the optimal clustering method for MZ delineation, and the input data should be normalized (range normalization). Feature selection was also shown to be positively effective. Furthermore, we proposed an algorithm based on DBSCAN for smoothing the MZs maps to allow smooth actuating during variable rate application by agricultural machinery. Finally, the whole process of MZ delineation was integrated in a clustering and smoothing pipeline (CaSP), which automatically performs the following steps sequentially: (1) range normalization, (2) feature selection based on cross-correlation analysis, (3) *k*-means clustering, and (4) smoothing. It is recommended to adopt the developed platform for automatic MZ delineation for variable rate applications of farming inputs.

## 1. Introduction

Traditional agricultural practices consider fields as homogeneous management units, under which farm operations assume no within-field variability in the soil or crop. However, agricultural soils are often extremely variable in space and time, and understanding its variability is essential to successfully manage farming inputs site-specifically and dynamically at a field scale [1]. To address within-field variability, variable management solutions are adopted using precision agriculture (PA) technologies, which aim at the site-specific application of farm inputs (e.g., seeds, fertilizers, manure, pesticides, and water) according to the soil and crop requirements [2]. Variable rate applications—also referred to as site-specific applications—are implemented in practice as map-based, sensor-based, or a combination of both approaches [3]. For both map-based and map-sensor-based solutions, the within-field variability should be classified into a few zones with similar characteristics. In fact, the most widely used approach to manage the variability of fields concerns the use of management zones (MZs) [4]. MZs are sub-areas of a field that have a relatively homogeneous combination of yield-limiting factors with respect to soil–landscape attributes [5], for which a single rate of a specific crop input is appropriate to maximize outputs such as yield and yield quality [6]. The accurate delineation of MZ maps is the key requirement for the successful implementation of map-based and map-sensor-based variable rate applications.

Several approaches are introduced in [2] to delineate MZs by the data fusion of several layers of information including farmer’s knowledge, terrain attributes, weather conditions, soil type, yield data from several seasons, crop growth characteristics, and soil properties. Indeed, MZ delineation considers the variables that are correlated to the yield, since the goal is to maximize the yield [7]. Guerrero et al. in [8] have shown that involving more layers of information in MZ delineation provides more robust results in terms of improving yield. De Benedetto et al. [9] delineated homogeneous areas by data fusion of electromagnetic induction sensor, a ground penetration radar, and remote sensing satellite hyperspectral images. Fleming et al. [10] combined the data of soil organic matter, clay, nitrate, potassium, zinc, electrical conductivity, and corn yield for variable-rate fertilization purposes. Pantazi et al. [4] proposed to delineate MZ maps by means of self-organizing clustering using soil data collected by an on-line soil sensing platform [11], crop normalized differential vegetation index (NDVI) of satellite imagery, and historical yield data.

Data fusion *potentially* reduces the prediction variance and hence improves the prediction precision [12]. However, when fusing different kinds of data, double counting the same information given by those data may degrade the overall performance [13,14,15]. Furthermore, as pointed out by Schenatto et al. [16], different kinds of data with different values’ ranges can impact the MZ delineation and result in favor of just a few of the involving data. Accordingly, we analyzed the impacts of feature selection and data normalization as solutions to correlation and inconsistent data ranges, respectively, in this paper.

To characterize the within-field variability, different sampling methods can be used. The most common method of soil sampling in a field is grid sampling mostly adopted to determine the chemical and physical properties [17]. Another sampling method is the use of proximal soil-sensing technologies to collect high-density data of 1000–2000 reading per ha, which introduces complexity and sources of errors, particularly when the resolution in one direction is much different than the resolution in the other direction. Examples include the online data collected by the visible and nearinfrared (vis-NIR) spectroscopy [18,19], where a resolution of 1 m by 10 m is very common, introducing complexity during interpolation and clustering.

MZ delineation can be considered as a data-mining problem, as it contemplates either classifying or clustering the field into a number of contiguous areas [20]. For MZ delineation, numerous clustering algorithms—such as *k*-mean, mean shift, fuzzy C-means (FCM), hierarchical clustering, density-based spatial clustering of applications with noise (DBSCAN), and particle swarm algorithm (PSO) [21,22,23]—have been already adopted. Recently, a deep-learning-based algorithm has also been examined by Javadi et al. [24]. However, all the examined algorithms have their own peculiarities in terms of features and efficiency (Karkra1 et al., 2020), since clustering is a complex task owing to the large number of interrelated parameters, resulting in a nonlinear problem. One source of nonlinearity stems from the inconsistent sampling resolution, which is common with online proximal soil sensing.

Clustering techniques are mostly unsupervised and attempt to explore the inherent structure of the data, often in terms of Euclidean distance. Different normalization methods were evaluated [16] for MZ delineation, without any clear discussion on why the data should be normalized. On the other hand, an apple orchard was delineated into MZs in [25] using a geostatistics method, in which the spatial correlation of data is taken into account. The spatial correlation has been also suggested to be considered in [26], where multicollocated cokriging was used for variable-rate fertigation. Indeed, soil and crop properties in agricultural fields generally present spatial dependence; hence, it is important to use geostatistical methods (kriging interpolation after semivariogram analysis) where soil or crop properties are considered as random regionalized variables, and the gradual geographical variation is described by a spatial covariance function [25,26]. Most of the papers concerning MZ delineation have not explicitly discussed the inclusion of the geographical coordinates of the data. However, the coordinates data were explicitly used in [9,25,27,28,29,30]. However, so far, there is no consensus not only on the clustering method but also the format of input data and inclusion or exclusion of spatial correlation and locations. This is particularly true when at least one layer of data involved in the clustering is collected at inconsistent sampling resolution over space, such as the example of the online vis-NIR sensor.

In this paper, we evaluated five clustering methods, namely, *k*-means, FCM, shift mean, hierarchical, and DBSCAN, in MZ delineation of five fields with different sizes in different regions of Belgium. The goal was to determine the optimal clustering method and data inputs for the delineation of MZs, using online collected soil data with inconsistent spatial resolution in addition to yield and crop data obtained from processing the data of satellite Sentinel2. Furthermore, we proposed a clustering and smoothing pipeline (CaSP) for MZ delineation, which gives a smoothed scheme of MZs and is applicable in practice by the variable rate agricultural machinery. We examined the performance of the proposed CaSP in the delineation of MZs maps in all the five studied fields using spatial statistical indicators.

## 2. Materials and Methods

**Figure 1:**
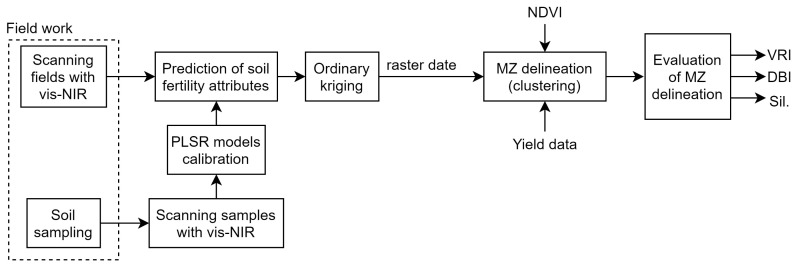
The flowchart of the evaluation steps different management zone (MZ) delineation schemes based on on-line collected soil fertility attributes, normalized difference vegetation index (NDVI), and yield. Different MZ delineation schemes were evaluated in terms of variance reduction index (VRI), Davies–Boulding index (DBI), and Silhouette index (Sil.). The soil fertility attributes were predicted based on visible-near-infrared (vis-NIR) readings.

The flowchart of the steps performed in this study for the different MZ delineation schemes is depicted in Figure 1. Each step is elaborated in what follows.

### 2.1. Experimental Sites

**Figure 2:**
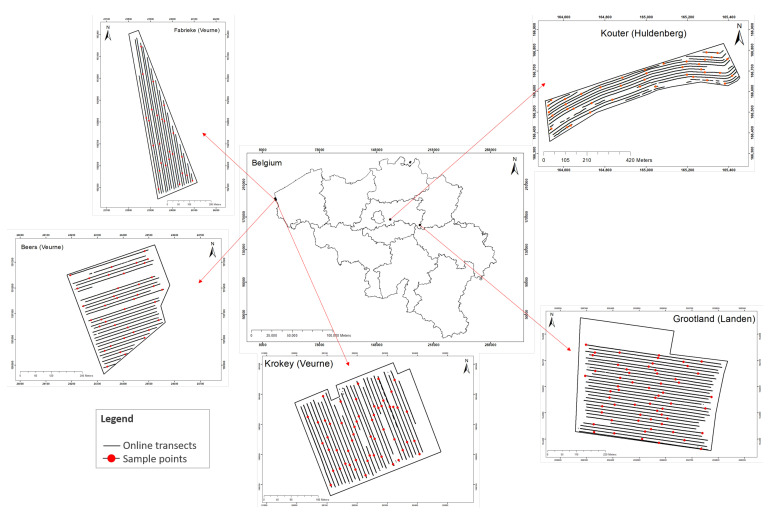
Locations of the five experimental sites in Flanders, Belgium, along with the online scanning lines and the locations of the random soil sampling points in Fabrieke, Beers, Krokey, Kouter, and Grootland.

The soil fertility attributes, normalized differential vegetation index (NDVI), and yield data of five commercial fields in the Flanders region in Belgium were used in this study (Figure 2). The soil fertility attributes included moisture content (MC), organic carbon (OC), calcium (Ca), cation-exchangeable content (CEC), exchangeable potassium (K), magnesium (Mg), sodium (Na), pH, and exchangeable phosphorus (P). The study fields consisted of a 21 ha field in Landen called Grootland (N $50^{\circ }47^{\prime }22.5^{\prime \prime }$, E $5^{\circ }6^{\prime }48.8^{\prime \prime }$), an 12 ha field in Huldenberg named Kouter (N $50^{\circ }48^{\prime }38.9^{\prime \prime }$, E $4^{\circ }34^{\prime }50.1^{\prime \prime }$), and three fields in Veurne: a 12 ha field called Beers (N $51^{\circ }1^{\prime }1.4^{\prime \prime }$, E $2^{\circ }34^{\prime }42.8^{\prime \prime }$), a 8 ha field named Fabrieke (N $51^{\circ }1^{\prime }53.9^{\prime \prime }$, E $2^{\circ }34^{\prime }16.9^{\prime \prime }$), and a 12 ha field designated as Krokey (N $50^{\circ }59^{\prime }58.3^{\prime \prime }$, E $2^{\circ }32^{\prime }52.1^{\prime \prime }$). The results of a soil texture analysis determined by means of the Robinson–Kohn pipette method (ISO 11277) indicated that soil in Grootland, Krokey, and Kouter was a silty loam, in Beers was a sandy loam, and in Fabrieke was a loam according to the United State Department of Agriculture (USDA) classification (Table 1). This region registers an annual average temperature of 10.6 ${}^{\circ }$C and a monthly average precipitation of 39.68 mm. The fields have an annual crop rotation of wheat, barley, oilseed rape, sugar beet, and potatoes with a short duration autumn cover crop.

### 2.2. Data Acquisition

**Figure 3:**
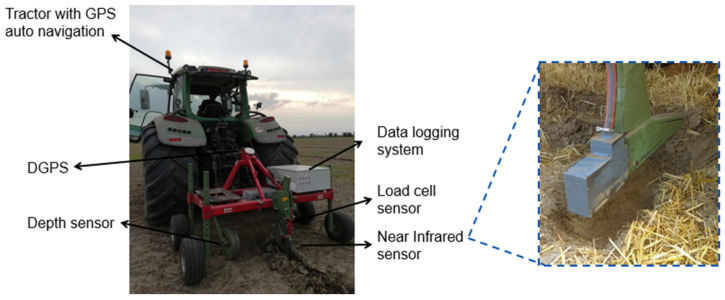
The multiple-sensor platform used for collecting soil data. DGPS: differential global positioning system.

Soil data were obtained by scanning the fields with an online multi-sensor platform, as shown in Figure 3, which was designed and developed by Mouazen [11] as discussed in [8,31]. The platform included a vis-NIR spectroscopy sensor (Tec5 Ag, Germany) with a measurement range of 305–1700 nm. The platform is attached to a tractor by means of the three-point hitch and pulled along parallel lines at a distance of 12 m between neighboring lines and at an average speed of 3 km/h. By creating a trench with a subsoiler, the platform is capable of collecting the vis-NIR soil spectra at 15–25 cm depth every second. It includes a differential global positioning system (DGPS) with RTK correction and a position accuracy of ±0.2 m (version CFX-750, Trimble, Sunnyvale, CA, USA) and a datalogger (Compact Rio 9082, National Instruments, USA) to acquire and store the collected soil spectra and DGPS readings at 1 Hz, using a custom-built Labview software (National Instruments, USA). Kouter, Beers, and Grootland were scanned in 2018, and Fabrieke and Krokey were scanned in 2019 after harvest of the previous crops (Figure 2).

In addition to the vis-NIR data, the NDVI data were obtained from the processed data of the Sentinel2 satellite imagery for Beers, Fabrieke, Krokey, and Grootland. Some soil attributes can be estimated using satellite data [32,33]; however, this was not the case in this study, since the accuracy of satellite data can degrade the high accuracy of soil attribute estimation models derived from vis-NIR spectra [13,34,35]. For the Kouter field, high-resolution NDVI data were collected using six Green Seeker sensors installed on a liquid fertilizer sprayer. To use NDVI data obtained from the satellite imagery and the Green Seeker sensor, extra data processing was performed. First, a kriging interpolation using the NDVI values along each field was performed; then, a common grid of 5×5 m was created, and finally, NDVI values were extracted for each pair of coordinates in the common grid. The yields data of cereal crops in the previous season in each field were collected using combine harvesters equipped with yield sensors providing high-resolution yield data (in Kouter field: John Deere W550, in Grootland field: Claas Lexion 740 with yield monitoring with Quantimeter, and in Fabrieke and Beers fields: Class Lexion 760 with yield monitoring with Quantimeter).

### 2.3. Modeling of Visible and Near-Infrared Spectra

In addition to the online measurements, random soil samples were collected manually from each field (Table 1) with the aim to build prediction models for soil attributes (pH, Ca, Mg, MC, OC, P, CEC, K, and Na), similar to what was explained in [3] (pp. 1–38) and [13,34]. A total of 155, 179, and 121 soil samples were collected from different fields in three farms, and these were used to develop three groups of models for Huldenberg, Landen, and Veurne farms, respectively. Cross-validation by using the leave-one-out technique was possible for the Kouter field (in Huldenberg farm), since limited data were available from this field to support independent validation (40 samples). In the other fields, the entire dataset was divided into calibration (70%) and validation (30%) sets. Afterwards, pre-treatment algorithms were applied to enhance the accuracy of the prediction models. These algorithms included removal of the spectral shift at 1045 nm [36], cutting noisy parts at the edges of the spectra, moving average to reduce spectral noise, standard normal variate transformation [37] or normalization, a Savitzky–Golay first derivative and a Savitzky–Golay smoothing. Finally, after performing a principal component analysis (PCA) to investigate the similarities or dissimilarities in the spectra, we developed partial least squares regression (PLSR) models for the prediction of the soil attributes in RStudio version 1.1.463 (RStudio Inc., Boston, MA, USA) with open-source libraries [38].

### 2.4. Mapping of Online Measured Soil Properties

The developed PLSR calibration models were used for estimating the soil attributes using the online collected spectra in the five fields. Then, since the attributes of soil in a field are spatial correlated [39], high-resolution maps of the soil attributes were obtained using ordinary kriging [40]. In ordinary kriging, an estimation of any attribute in any point is given by a linear combination of the available measurements while the weights of the linear combination are obtained from semivariograms [41]. After the kriging interpolation, all attributes were resampled to a common grid of 5×5 m and a pair of geographical coordinates was calculated for each of the grid points.

It is worth mentioning that ordinary kriging was adopted in this study since, despite simple kriging, it does not assume prior knowledge of the mean and covariance of the attributes in a land, and hence, it is the most common kriging approach in the literature of management zone delineation [2,16]. Other types of kriging, such as block kriging, universal kriging, and indicator-based kriging, also exist, in which attempts to improve the interpolation performance result in the cost of more computational power demand. However, since the focus of this study was not on kriging, we resorted to the most common approach.

### 2.5. Overview of Clustering Algorithms

In this study, the performance of five clustering algorithms was evaluated in different scenarios. The clustering algorithms used were unsupervised since the data, i.e., the geo-referenced soil attributes, were not labeled. The algorithms have briefly been discussed in the following subsections. In discussing the clustering algorithms, $x_{i}$ denotes the *i*th *d*-dimensional input data, i∈{1,…,n}, $c_{i}$ denotes the cluster to which $x_{i}$ belongs, and $\mu_{j}$ is the centroid of cluster j∈{1,…,k} with *k* being the number of the clusters.

#### 2.5.1. *k*-Means

*k*-means divides the *n*-dimensional data into *k* categories with the objective to minimize the sum of the within-cluster variances. While simple, it is considered as an efficient clustering algorithms in many data analysis applications. It needs *k* to be defined and works as follows:

1. Randomly initialize *k* cluster centroids $\mu_{1},\ldots ,\mu_{k}\in R^{d}$.
2. For i∈{1,…,n}, update:
  $$c_{i}=\arg\min_{j}{∥x_{i}-\mu_{j}∥}^{2}\;.$$
3. For j∈{1,…,k}, update $\mu_{j}=$ centroid of the data of cluster *j*.
4. Repeat steps 2 and 3 for a specified number of iterations (or until convergence).

#### 2.5.2. Fuzzy C-Means (FCM)

FCM works similarly to *k*-means. The only difference is that it aims at minimizing the weighted sum of the within-cluster variances. The weights define the clustering fuzziness. Indeed, FCM does not strictly assign each point to a specific cluster. Instead, the cluster membership is fuzzy. The algorithm works as discussed below.

Parameters: *k*, *m* (fuzziness coefficient—a real number greater than 1)

FCM algorithm:

1. Randomly initialize *k* cluster centroids $\mu_{1},\ldots ,\mu_{k}\in R^{d}$.
2. For i∈{1,…,n} and j∈{1,…,k}, update:
  $$u_{ij}=\frac{1}{\sum_{l=1}^{k}{\left(\frac{∥x_{i}-\mu_{j}∥}{∥x_{i}-\mu_{l}∥}\right)}^{\frac{2}{m-1}}}\;.$$
3. For j∈{1,…,k}, update:
  $$\mu_{j}=\frac{\sum_{i=1}^{n}u_{ij}^{m}x_{i}}{\sum_{i=1}^{n}u_{ij}^{m}}\;.$$
4. Repeat steps 2 and 3 for a specified number of iteration (or until convergence).
5. After the algorithm stops, each point *i* joins the cluster with the highest $u_{ij}$ value.

#### 2.5.3. Mean Shift

Mean shift is a density-based mode-seeking algorithm. It tries to first estimate the density of the data by using a kernel and then looks for the modes of the distribution. In order to find the modes, it iteratively moves each point to its denser neighborhood. Mean shift is a non-parametric clustering algorithm, meaning that it does not need the number of clusters to be specified in advance. Instead, it tries to find the number of clustering according to the density of the data. The algorithm steps are as follows.

Parameters: *h* (the kernel bandwidth—note that a kernel should be chosen in advance. The mostly used kernel is the Gaussian kernel).

Mean shift algorithm:

1. Initialize seeds set S for calculating the density
  $$f\left(x\right)=\sum_{x_{i}\in S}K\left(x-x_{i}\right)\;,$$
  where K(.) is a kernel function.
2. For each seed s∈S, calculate the mean shift:
  $$m\left(s\right)=\frac{\sum_{x_{i}\in s}K\left(x_{i}-s\right)x_{i}}{\sum_{x_{i}\in N\left(s\right)K\left(x_{i}-s\right)}}\;,$$
  where N(s) denotes the neighborhood of s.
3. For each seed s∈S, update s=m(s).
4. Repeat steps 2 and 3 for a specified number of iterations (or until convergence).
5. After the algorithm stops, the modes are considered as the centroids of the clusters, and each point joins to the closest mode.

#### 2.5.4. Hierarchical Clustering

The hierarchical clustering algorithm seeks to build a hierarchy of clusters based on either of two approaches: agglomerative or divisive. Agglomerative is a bottom–up approach based on which pairs of clusters are merged together in order to build up the hierarchy. On the other hand, the divisive approach is a top–down method that starts from one cluster including all data and then splits the cluster recursively. In this paper, the agglomerative hierarchical clustering was adopted. The algorithm receives *k* as the input parameter and works as follows:

1. Assign all points an individual cluster number.
2. Merge points with the smallest distance. In other words, points with smallest distance join the same cluster.
3. Repeat step 2 until *k* clusters are obtained.

#### 2.5.5. Density-Based Spatial Clustering of Applications with Noise (DBSCAN)

DBSCAN, similar to mean shift, is a non-parametric clustering algorithm. In other words, the number of clusters, *k*, does not need to be specified for it. Instead, it reaches a number of clusters based on the density of data and two parameters discussed below.

Parameters: ϵ (the neighborhood distance), $m_{p}$ (minimum number of data points to define a cluster)

DBSCAN algorithm:

1. Select a random data point.
2. If the number of the neighbors is less than $m_{p}$, the point is marked as an outlier with label −1.
3. If the number of the neighbors is more than or equal to $m_{p}$, the point and its neighbors establish a cluster.
4. Repeat step 3 for all points within the established cluster. In other words, for all joined points, check their neighbor points and join their neighbors to the established cluster.
5. From the remaining points that have not yet been met, select a random data point. Repeat steps 2 to 5 until all data points are met.

### 2.6. Feature Selection (Data Decrease)

Among the soil attributes used for MZ delineation, OC and MC have direct signatures in the spectral characteristics in the vis-NIR range, while other attributes are estimated indirectly based on their correlation with MC and OC [19]. Applying correlated features to clustering models imposes computational burden and might degrade the clustering quality. Accordingly, we studied the effect of feature selection by cross-correlation analysis. To this end, the cross-correlation of the data was computed using Pearson correlation in order to remove highly correlated layers. The removal of highly correlated data before clustering in MZ delineation applications has also been recommended by [16,42].

### 2.7. Clustering Scenarios

We evaluated the above-mentioned clustering algorithms in different scenarios listed in Table 2. In all scenarios, except in kmeans-nn-nc, data are normalized. For normalization, the data ranges were scaled into an interval between 0 and 1. In other words, range normalization was applied to the data, since it has been shown to be the most effective normalization method in MZ delineation applications [16].

### 2.8. Evaluation of Clustering Algorithms

Since the clustering algorithms used in this study were unsupervised, it was not possible to evaluate their performances by comparing the clustering results against true labels. Instead, there exist heuristic metrics to assess the quality of unsupervised clustering; however, these metrics do not measure the validity of the model’s predictions. In order to choose the most appropriate clustering scheme for MZ delineation, we adopted three metrics, namely, Davies–Bouldin index (DBI) [43], Silhouette index [44], and variation reduction index (VRI) [16,45].

Considering *k* clusters, DBI is computed by [43]:

(1) $$DBI=\frac{1}{k}\sum_{i=1}^{k}\max\;\frac{s_{i}+s_{j}}{d_{ij}},$$

where $s_{i}$ is the average distance of the data of cluster *i* from its center and $d_{ij}$ denotes the distance between the centers of cluster *i* and cluster *j*. In fact, the intuition of DBI is that the clustering schemes with denser clusters that are further from each other are better. DBI is always positive, and its fewer values indicate better clustering quality and vice versa.

The Silhouette index quantifies the clustering quality by defining how well each data point has been assigned to its own cluster. Considering *k* clusters, the Silhouette index for data *i* is given by [44]:

(2) $$s\left(i\right)=\frac{b(i)-a(i)}{\max\{a(i),b(i)\}},$$

where $a\left(i\right)=\frac{1}{\left|C_{i}\right|-1}\sum_{j\in C_{i},j\neq i}d\left(i,j\right)$ and $b\left(i\right)=\min_{l\neq i}\frac{1}{\left|C_{l}\right|}\sum_{j\in C_{l}}d\left(i,j\right)$, respectively, indicate the similarity of data *i* to its own cluster and its dissimilarity to other clusters with $\left|C_{l}\right|$ and d(i,j) being the size of cluster *l* and the distance between data *i* and *j*, respectively. The overall Silhouette index is computed by averaging the indices of all data. Silhouette values range between −1 and 1 with more values indicating better clustering quality and vice versa.

DBI and Silhouette indices have emerged from the machine learning (ML) context and are applicable on any application, including those in precision agriculture. We also adopted VRI introduced by [45] specifically for evaluating the quality of MZ delineation. The VRI rational is that the variance of the soil attributes within the MZs should be less than their overall variance. The more the within-cluster variances decrease, the better the clustering quality. The VRI for soil attribute θ is given by [45]:

$$VRI_{\theta }=\left(1-\frac{\sum_{i=1}^{k}A_{i}v_{C_{i}}^{\theta }}{v^{\theta }}\right)\times 100\%,$$

in which $A_{i}$ is the proportion of the area covered by cluster *i*, $v^{\theta }$ is the overall variance of the soil attribute θ, and $v_{C_{i}}^{\theta }$ denotes its variance within cluster *i*. More values of VRI implicate better MZ delineation quality and vice versa. In this paper, the overall VRI is obtained by averaging the VRI of all studied soil attributes.

### 2.9. Clustering and Smoothing Pipeline (CaSP) for Management Zone Delineation

**Figure 4:**
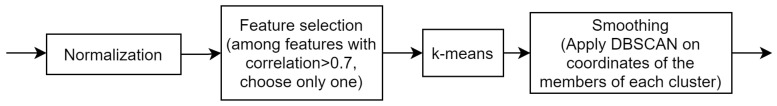
The clustering and smoothing pipeline (CaSP) for the management zone delineation approach of the current work.

After evaluation of different clustering methods in different scenarios, the most appropriate method was specified. Nevertheless, the clustering results usually include small parts of a cluster located apart within another cluster. Let us refer to these small parts as islands, since this is what they really look like. On the other hand, the clustering results are used in form of a recommendation map for variable-rate application of farming inputs such as fertilizers [8], manure [46], and seeds [47], for which the actuators of the agricultural machines cannot respond to the small modification needed in the applied rate due to these small islands. Therefore, it was necessary to introduce an additional smoothing step to the MZ maps in order to make them appropriate for variable-rate implementation in practice, as shown in Figure 4. To this end, we used the DBSCAN algorithm and applied it to just the geographic coordinates of the data points in each cluster, since DBSCAN is in nature capable of discovering the islands and single apart data points (as outliers) [48], as discussed in Section 2.5. The pseudocode of the smoothing algorithm has been shown in Algorithm 1. This algorithm takes as input the geographic locations of the data in a Cartesian system (i.e., xy coordinate) together with their corresponding labels and the island size. The xy coordinates of the data of each cluster are clustered by DBSCAN. Then, the outliers will join to the cluster of their closest data point. If more than one cluster is obtained, it means that the cluster includes two or more separate parts. Then, the algorithm joins the parts smaller than island size to another cluster according to the majority rule. More specifically, the algorithm first finds the edge points of the island area. Then, the label of the island data is changed to the majority of the labels of the neighbors of the edge points. The overall clustering and smoothing pipeline (CaSP) for MZ delineation is shown in Figure 4.
**Algorithm 1:** Smoothing algorithm
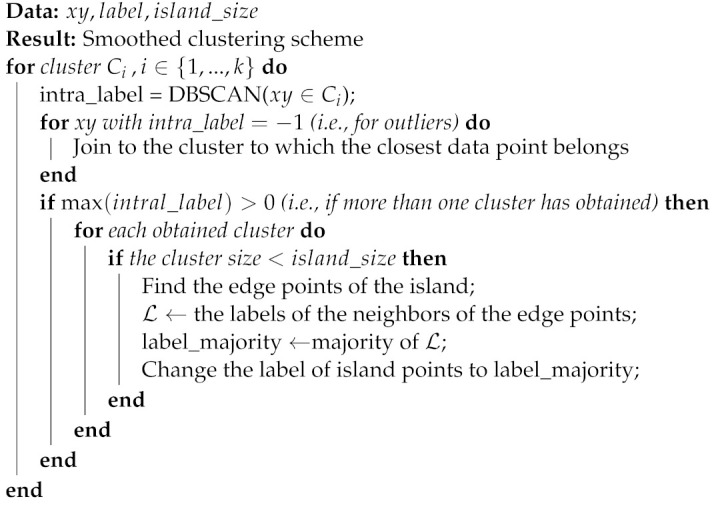


## 3. Results and Discussion

### 3.1. Evaluation of Clustering Algorithms

Mean shift and DBSCAN are non-parametric methods, meaning that the number of MZs is not needed to be specified. However, we evaluated the performance of *k*-means, FCM, and hierarchical methods for dividing the fields into four MZs (i.e., k=4). For FCM, in addition to *k*, the fuzziness coefficient *m* needed to be specified. We used m=2, since this value has been suggested in most applications [49]. Mean shift needed just the kernel bandwidth *h* to be set, for which we used the related function in the Sklearn package of Python (version 1.0.2) [50]. This function gave a value around h=0.41. For DBSCAN, we considered $m_{p}=3$ and set ϵ as the mean of the spatial scanning resolution.

The evaluation results of the clustering algorithms in the studied scenarios have been listed in Table 3. Recall that higher values of the Silhouette index and VRI and smaller DBI values indicate better clustering performance. According to their definitions in Equations (1) and (2), DBI and the Silhouette index are not defined for cases with just one cluster, for which VRI is zero. In the studied scenarios, mean shift and DBSCAN gave just one cluster in some cases, whose indices have been specified by n.v. (no value) in Table 3.

The data were normalized in all scenarios except in kmeans-nn-nc. Although DBI and the Silhouette index suggest this scenario as the best, its VRI values are poor. As will be elaborated later, normalization is essential in clustering, since ignoring it will weigh more on the data with large values and neglect data with small values (e.g., weighs more on Ca and neglects OC and pH). Accordingly, the first scenario (e.g., no normalization) is not recommended. Among other scenarios, VRI has always suggested to use *k*-means. The Silhouette index agrees with VRI in Kouter, Beers, and Fabrieke, while it suggests mean shift for Krokey and Grooteland. On the other hand, DBI has the best values for either mean shifting or DBSCAN.

**Figure 5:**
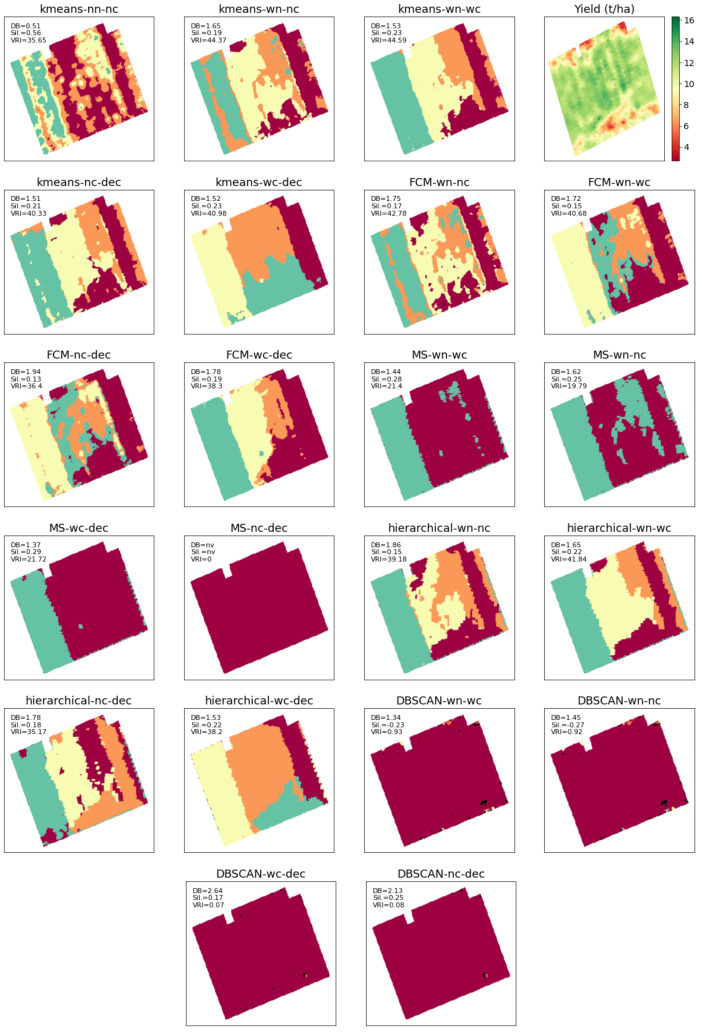
Comparison of different clustering schemes in delineation of management zones, shown for field Krokey as an example. When not explicitly mentioned in the scheme title, normalization is included. The clustering performances are evaluated in terms of Davies–Bouldin score (DB), Silhouette score (Sil.), and variance reduction index (VRI).

A close examination is needed into the MZs obtained by different scenarios in order to conclude on the format of the input data, i.e., normalizing or not, inclusion or exclusion of the coordinates, and whether or not to apply feature selection. For the sake of brevity, we describe the MZs results and the yield map for just field Krokey—as shown in Figure 5—since this field has more variability compared to other fields. As seen, DBSCAN gave just one cluster with several points as outliers. Indeed, DBSCAN performs clustering based on density and considers a cluster as unique as long as it is dense. Density-based clustering algorithms are helpful in object detection in machine vision applications. Since the focus in PA applications is on within-field variability, DBSCAN does not perform desirably and has to be excluded. This argument applies to mean shift as well and also has to be excluded for variable rate applications (Figure 5). Compared to DBSCAN, mean shift has two more disadvantages: (1) it demands a high computational power and (2) it is very sensitive to its parameter *h*.

It is worth noting that as in case of Krokey, DBSCAN and mean shift are shown to be not suitable for the other three fields of this study due to the same conclusion drawn above. FCM works similar to *k*-means but needs an additional hyperparameter as the fuzziness coefficient to be specified while there is no clue on how to set this parameter for variable rate applications. This makes *k*-means as the best method for clustering, whose results need to be analyzed further. Among the *k*-means results, *k*-means with no coordinate data (i.e., just using the soil attributes) after feature selection (i.e., kmeans-nc-dec) visually shows more correlation with the yield map, and this observation was also confirmed by the farmer. Revisiting Table 3 indicates that the VRI and Silhouette indices of kmeans-nc-dec are, if not maximum, among the highest values throughout all the five study fields. Its DBI has also the small values compared to other scenarios. In the Fabrieke case, after excluding kmeans-nn-nc, all the three indices suggest using kmeans-nc-dec. In Kouter, both DBI and the Silhouette index suggest kmeans-nc-dec, for which VRI is also very close to its maximum value among *k*-means scenarios with normalized inputs. The values of the indices for the *k*-means scenarios are very close to each other in the other two fields, which allow drawing the same conclusion as that of Krokey that Kmeans-nc-dec is the best performing scenario. This is particularly true as this scenario has performed much better than all the other *k*-means solutions in Fabrieke. However, the kmeans-wn-nc shows comparable performance indicators to that of the kmeans-nc-dec, although its performance deteriorated significantly for Fabrieke. This is the reason why it is concluded that MZ delineation using Kmeans-nc-dec scenario would provide the most stable solution in general.

From the computational point of view, kmeans-nc-dec is also optimal, since excluding coordinates decreases the data dimension, while the dimension is further reduced after feature selection. It is worth noting that since some of the soil attributes are spatially correlated (e.g., Ca and CEC), including spatial data in clustering is generally recommended, as they were explicitly considered in similar applications [9,25,27,28,29,30]. Since we used the raster data after spatial interpolation using kriging, the spatial correlation has been taken into consideration once, so that reconsidering it one more time means overweighing the spatial information.

**Figure 6:**
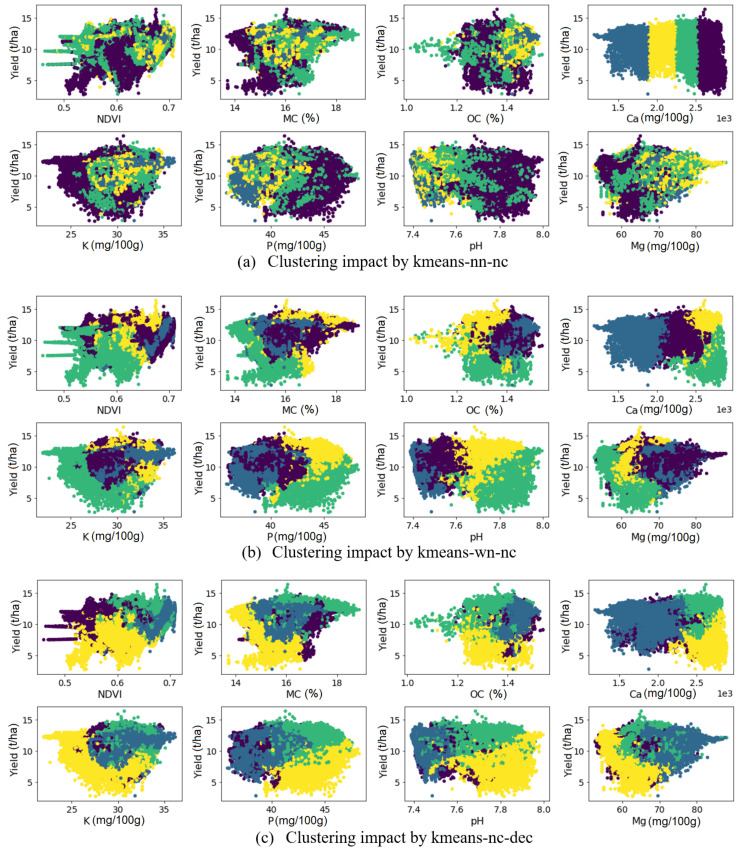

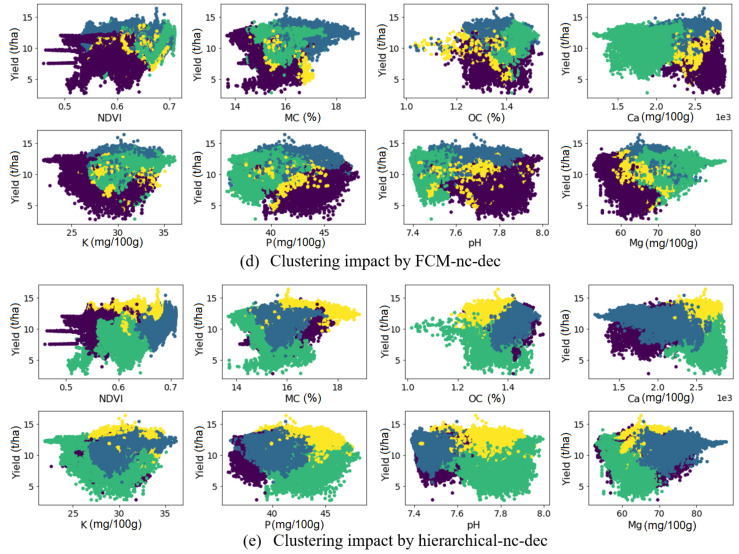
The impact of the clustering schemes on the scatter plot of yield vs. NDVI, and other soil attributes.

Figure 6 demonstrates the scatter plot of yield vs. the soil attributes and crop NDVI in selected clustering scenarios shown for field Krokey, as an example. Figure 6a highlights the importance of normalizing the data. Since the clustering algorithms use (Euclidean) distance for establishing clusters, the data should be normalized in order for all soil attributes to impact the distance equally; otherwise, only the data with large values will produce the clustering outcome. As seen in Figure 6a, the clustering outcome was affected mainly by Ca variability, because the Ca values were much larger than the other attributes (Figure 6a). Its great overlap between the four clusters is depicted for the other soil attributes. The scatter plots in the other scenarios depict that clustering has taken all the input variables equally since they were normalized; however, each scenario gave different clustering outcomes, which is attributed to applying different algorithms on the data. After normalization, the overlap between the four different clusters was greatly reduced, and a clear separation of classes can be observed not only for Ca but also for the other soil attributes.

### 3.2. Evaluation of MZ Delineation by CaSP

**Figure 7:**
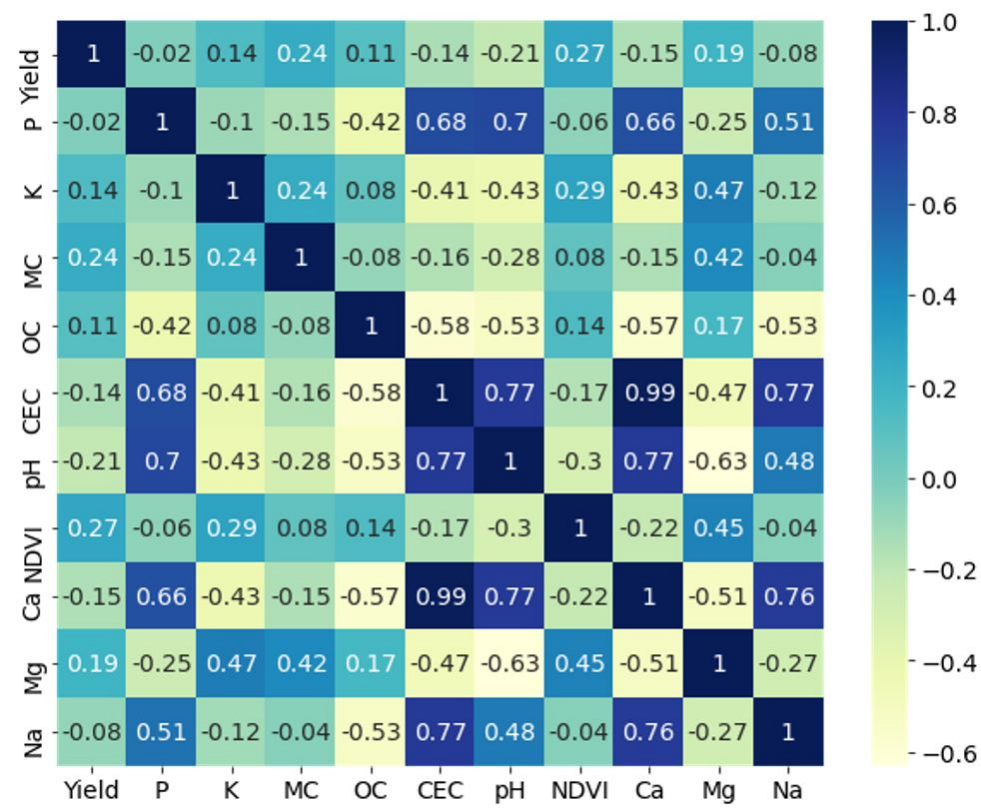
The cross-correlation (Pearson correlation) matrix of the soil attributes in field Krokey.

Feature selection by covariance analysis has been recommended by [16,42]. Figure 7 shows the cross-correlation matrix of the soil attributes for field Krokey as an example. It can be observed that there were high correlations (>0.7) among the soil Ca, CEC, Na, and pH, except between pH and Na, where a correlation of 0.48 was observed. Therefore, Ca, Na, and pH were removed from the analysis, since CEC was given a higher priority from a soil fertility perspective [51,52].

**Figure 8:**
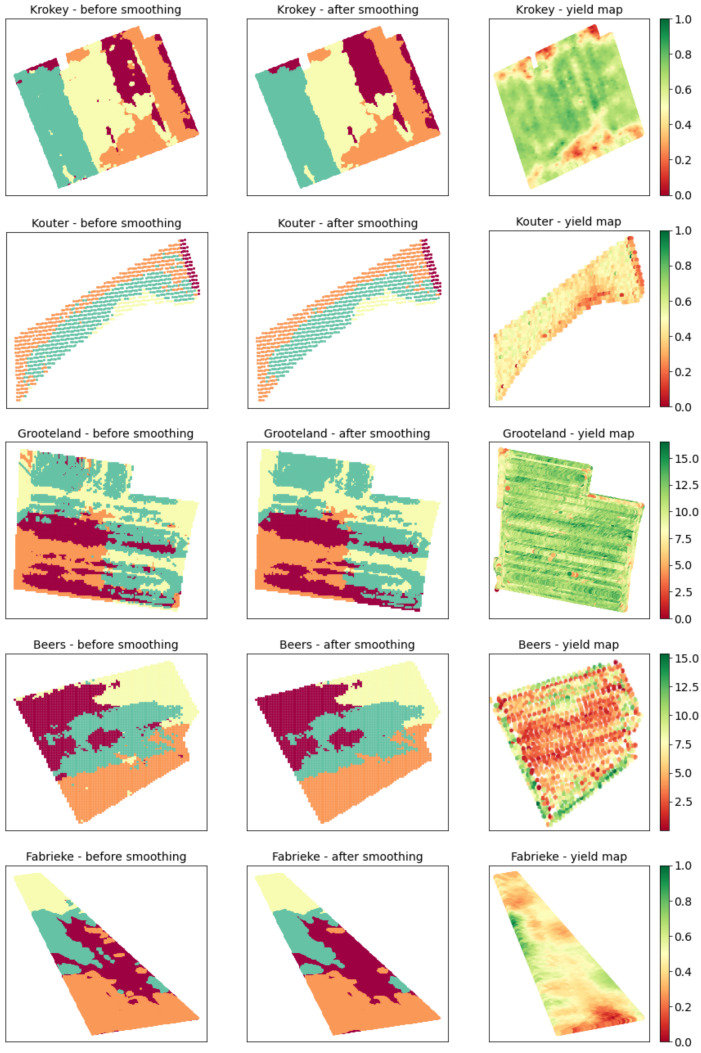
The clustering results before and after smoothing and comparing them with the yield maps in the study fields. The clustering schemes in the middle column are the outcome of management zone delineation by clustering and smoothing pipeline (CaSP).

The clustering results obtained by the proposed CaSP MZ delineation scheme has been illustrated in Figure 8, where MZ maps delineated with and without smoothing were compared. As can be seen, smoothing has appropriately filtered small apart areas (islands), which cannot be accounted for in practice during variable rate applications. While varying the rate of farming inputs using agricultural machinery equipped with PA control-enabling technology, it is necessary that the size of the machinery active control unit is smaller or equal to the smallest islands, i.e., they have to support high-resolution control, which is costly. In case the islands are smaller than the agricultural machinery size, then the variable rate will not be implementable in practice. The filter designed in this study is flexible and allows the filtering of islands with different sizes (Figure 4). However, removing very large-size islands can lead to ignoring important fertility zones in the field, for which the agricultural machine can respond to correctly during field operations. This feature allows for the implementation of different agricultural machinery during variable rate applications, each of which would require smoothing islands of different sizes to match the size of the machine to be used.

As seen in Figure 8, the MZ map given by *k*-means for Krokey shows an appropriate but partial visual correlation with the yield map. Interestingly, the MZ map was able to capture the fringe lane by the road (the lane in the right side of the MZ map). While smoothing the map has kept the similarity with the yield map, it has removed the very small island parts. The MZ maps of field Kouter with smaller measurement resolution also show a very good similarity with the yield map, which is a similarity that was better than that of Krokey field. In case of Grooteland, the MZ maps indicate high variability within the field, while the yield seems to be almost uniformly distributed over the field area. This is due to enforcing the algorithm to divide the field into four MZs, so that soil fertility attributes have the major contribution on the MZ map. However, exploring the MZ maps in more detail shows it still has some indicative correlations with the yield. Specifically, the management zone indicated by the beige color has captured the low-fertility zones of the field, and these are well correlated with areas with low values in the yield map. Here, smoothing was effective in making the MZ map more suitable for practical application. In the Beers and Fabrieke cases, there exists a good visual correlation between the MZ maps and yield. Specially, there was a low-fertility zone in the central region of the Beers field, which has been captured very well by CaSP. According to the above and when high-sampling resolution data on soil and crop are considered, we recommend the CaSP based on *k*-means clustering for automatic delineation of MZ maps for the deployment of variable rate applications of farming inputs. The ideal solution should have the following successive steps of data processing: (1) range normalization, (2) feature selection based on cross-correlation analysis, (3) *k*-means clustering, and (4) smoothing by DBSCAN.

## 4. Conclusions

In this paper, five clustering algorithms were evaluated in different scenarios for MZ delineation in five arable farming fields, with the intention to evaluate their suitability for variable rate applications. The clustering algorithms included *k*-means, FCM, mean shift, hierarchical, and DBSCAN. These algorithms were evaluated in scenarios with and without range normalization, geographical coordinates, and feature selection. On-line measured soil fertility attributes (pH, Ca, Mg, Na, P, CEC, MC, K, and OC) at high sampling resolution, along with crop NDVI and the yield data, were used as input to the clustering algorithms. Spatial interpolation using ordinary kriging was carried out in order to get high-resolution data.

The results suggested *k*-means as the optimal clustering algorithm after normalizing and exclusion of the GPS coordinates. Nevertheless, it was noted that the coordinates should be ignored, since the spatial correlations of the data had been previously considered when the data were interpolated using ordinary kriging. In general, if the data are not interpolated using any spatial interpolation algorithms, it is recommended to include the coordinates in order to account for the spatial correlation among soil attributes. Furthermore, it was concluded that feature selection optimized after cross-correlation analysis improves the MZ delineation quality while reducing computational burden.

Moreover, a smoothing algorithm was proposed based on DBSCAN for filtering out small areas of a cluster within other clusters. Overall, an MZ delineation pipeline was proposed including the following steps: (1) range normalization, (2) feature selection by cross-correlation analysis, (3) *k*-means clustering, and (4) smoothing. The effectiveness of this pipeline—to which we referred to as CaSP, standing for clustering and smoothing pipeline—was demonstrated by the practical application of MZs concerning the machinery size during variable rate applications. Future study directions may include analysis of the effect of the accuracy of soil attributes predictions on clustering quality and also improving the efficiency of the feature selection operator.

## Figures and Tables

**Table 1:** Information of the spectral library used for the development of visible and near-infrared (vis-NIR) calibration models for three farms for the prediction of key soil properties using the online spectra collected with the online multi-sensor platform. Reprinted with permission from Ref. [11]. Copyright 2021 Elsevier.

Model	Field Name	% Clay	% Sand	% Silt	Soil Texture (USDA)	No. Samples	Total Samples
Huldenberg	Kouter (Target field)	12.6	11.6	75.8	Silt Loam	40	155
	Duidelbergen	10.2	10.3	79.4	Silt Loam	24	
	Voor de Heeves	12.0	9.5	78.5	Silt Loam	43	
	Lange Weid	10.3	10.3	79.4	Silt Loam	48	
Landen	Grootland (Target field)	13.3	6.3	80.4	Silt Loam	60	179
	Gimgelomse	13.2	32.7	54.2	Silt Loam	38	
	Kattestraat	–	–	–	–	20	
	Dal	–	–	–	–	23	
	Bottelare ^1^	–	–	–	–	25	
	Thierry ^1^	–	–	–	–	13	
Veurne	Beers (Target field)	16.5	54.0	29.5	Sandy Loam	39	122
	Fabrieke (Target field)	16.2	37.8	46.0	Loam	25	
	Krokey (Target field) ^2^					54	
	Watermachine	14.5	51.6	33.9	Loam	20	
	Bottelare ^1^	–	–	–	–	25	
	Thierry ^1^	–	–	–	–	13	

^1^ These fields are located in Bottelare and Mouscron, respectively, but their data were used to improve the accuracy of models developed for the Landen and Veurne farm. ^2^ Krokey field was not included in de development of the Veurne model.

**Table 2:** The clustering scenarios evaluated in this study.

Clustering Scenario	Clustering Method and the Conditions of Its Input Data
kmeans-nn-nc ^1^	*k*-means, no data normalization, xy coordinate data not considered
kmeans-wn-nc	*k*-means, with data normalization, xy coordinate data not considered
kmeans-wn-wc	*k*-means, with data normalization, with xy coordinate data
kmeans-nc-dec	*k*-means, xy coordinate data not considered, data decreased
kmeans -wc-dec	*k*-means, with xy coordinate data, data decreased
FCM-wn-nc	FCM, with data normalization, xy coordinate data not considered
FCM-wn-wc	FCM, with data normalization, with xy coordinate data
FCM-nc-dec	FCM, xy coordinate data not considered, data decreased
FCM-wc-dec	FCM, with xy coordinate data, data decreased
MS-wn-nc	Mean shift, with data normalization, xy coordinate data not considered
MS-wn-wc	Mean shift, with data normalization, with xy coordinate data
MS-wc-dec	Mean shift, with xy coordinate data, data decreased
MS-nc-dec	Mean shift, xy coordinate data not considered, data decreased
hier-wn-nc	Hierarchical, with data normalization, xy coordinate data not considered
hierarchical-wn-wc	Hierarchical, with data normalization, with xy coordinate data
hierarchical-nc-dec	Hierarchical, xy coordinate data not considered, data decreased
hierarchical-wc-dec	Hierarchical, with xy coordinate data, data decreased
DBSCAN-wn-nc	DBSCAN, with data normalization, xy coordinate data not considered
DBSCAN-wn-wc	DBSCAN, with data normalization, with xy coordinate data
DBSCAN-wc-dec	DBSCAN, with xy coordinate data, data decreased
DBSCAN-nc-dec	DBSCAN, xy coordinate data not considered, data decreased

^1^ nn: no normalization; wn: with normalization; nc: no coordinate; wc: with coordinate; dec: decreased data; xy coordinate: cartesian coordinate.

**Table 3:** Evaluation of clustering methods including *k*-means, fuzzy C-means (FCM), mean shift (MS), hierarchical, and density-based spatial clustering of applications with noise (DBSCAN) in terms of Davies–Doublin index (DBI), Silhouette index (Sil.), and variance reduction index (VRI).

Field Name	Krokey			Kouter			Grooteland			Beers			Fabrieke		
**Score**	**DBI**	**Sil.**	**VRI**	**DBI**	**Sil.**	**VRI**	**DBI**	**Sil.**	**VRI**	**DBI**	**Sil.**	**VRI**	**DBI**	**Sil.**	**VRI**
km. ^1^-nn-nc	0.51	0.56	35.65	0.55	0.53	12.25	0.67	0.45	33.81	0.56	0.52	28.27	0.75	0.31	3.56
km.-wn-nc	1.65	0.19	44.37	1.55	0.25	33.42	1.40	0.21	47.82	1.33	0.25	45.35	1.43	0.20	23.56
km.-wn-wc	1.53	0.23	44.59	1.53	0.21	27.47	1.40	0.24	43.24	1.32	0.26	46.92	1.57	0.24	30.32
km.-nc-dec	1.51	0.21	40.33	1.33	0.27	33.31	1.45	0.20	45.86	1.36	0.24	45.19	1.21	0.31	48.50
km.-wc-dec	1.52	0.23	40.98	1.61	0.21	26.55	1.38	0.24	41.57	1.29	0.27	47.37	1.59	0.22	28.40
FCM-wn-nc	1.72	0.15	40.68	2.27	0.16	24.79	2.01	0.15	42.93	1.47	0.22	44.13	1.55	0.19	55.89
FCM-wn-wc	1.75	0.17	42.78	1.54	0.20	26.87	1.46	0.24	42.73	1.37	0.24	45.58	1.40	0.23	57.98
FCM-nc-dec	1.94	0.13	36.40	2.28	0.17	21.46	1.77	0.16	42.32	1.40	0.23	45.00	1.99	0.19	19.97
FCM-wc-dec	1.78	0.19	38.30	1.63	0.19	25.29	1.42	0.23	41.18	1.31	0.26	46.80	1.67	0.17	10.56
MS-wn-nc	1.44	0.28	21.40	1.63	0.20	25.29	1.42	0.23	41.18	1.31	0.25	46.80	1.40	0.23	58.28
MS-wn-wc	1.62	0.25	19.79	3.45	0.07	24.89	1.43	0.26	30.28	1.41	0.23	39.73	1.00	0.32	38.30
MS-wc-dec	1.37	0.29	21.72	1.47	0.02	20.75	1.69	0.22	21.45	1.32	0.28	37.78	1.43	0.10	24.67
MS-nc-dec	n.v.	n.v.	0	1.05	0.15	9.56	1.43	0.23	27.16	1.67	0.23	21.92	0.93	0.16	34.23
hier. ^2^-wn-nc	1.86	0.15	39.18	2.42	0.24	16.88	2.06	0.16	36.90	1.70	0.22	37.86	1.25	0.25	55.18
hier.-wn-wc	1.65	0.22	41.84	2.17	0.18	18.18	1.65	0.23	36.65	1.51	0.22	40.16	1.30	0.27	55.58
hier.-nc-dec	1.78	0.18	35.17	1.37	0.25	31.02	1.67	0.17	41.65	1.44	0.21	41.58	1.43	0.23	29.04
hier.-wc-dec	1.53	0.22	38.20	1.52	0.23	29.67	1.38	0.22	38.91	1.38	0.25	45.43	1.49	0.21	28.58
DBS. ^3^-wn-nc	1.34	-0.23	0.93	2.34	0.13	10.30	n.v.	n.v.	0	n.v.	n.v.	0	2.76	0.16	5.77
DBS.-wn-wc	1.45	-0.27	0.92	2.76	0.01	7.54	4.27	0.08	1.84	3.05	0.07	7.90	2.70	0.12	5.59
DBS.-wc-dec	2.64	0.17	0.07	1.99	0.20	10.88	1.28	0.20	0.47	0.85	0.06	1.10	2.21	0.18	14.29
DBS.-nc-dec	2.13	0.25	0.08	1.82	0.26	11.84	1.72	0.00	1.94	2.42	-0.1	4.43	2.03	0.23	14.11

^1^ kmeans. ^2^ hierarchical. ^3^ DBSCAN.

## Abbreviations

The following abbreviations are used in this manuscript:

Ca	Calcium
CaSP	Clustering and smoothing platform
CEC	Cation exchange capacity
DBI	Davies–Bouldin index
DBSCAN	Density-based spatial clustering of applications with noise
DGPS	Differential global positioning system
FCM	Fuzzy C-means
K	Exchangeable potassium
MC	Moisture content
Mg	Magnesium
MS	Mean-shift
MZ	Management zone
Na	Sodium
NDVI	Normalized difference vegetation index
OC	Organic carbon
P	Exchangeable phosphorous
PA	Precision agriculture
Sil.	Silhouette
vis-NIR	Visible-near-infrared
VRI	Variance reduction index
